# Multi-isocenter VMAT craniospinal irradiation using feasibility dose–volume histogram-guided auto-planning technique

**DOI:** 10.1093/jrr/rrad026

**Published:** 2023-05-04

**Authors:** Yun Zhang, Yuling Huang, Jiafan Lin, Shenggou Ding, Xiaochang Gong, Qiegen Liu, Changfei Gong

**Affiliations:** Department of Radiation Oncology, Jiangxi Cancer Hospital, 519 East Beijing Road, Qingshanhu District, Nanchang 330029, China; Department of Radiation Oncology, Jiangxi Cancer Hospital, 519 East Beijing Road, Qingshanhu District, Nanchang 330029, China; Department of Radiation Oncology, Jiangxi Cancer Hospital, 519 East Beijing Road, Qingshanhu District, Nanchang 330029, China; Department of Radiation Oncology, Jiangxi Cancer Hospital, 519 East Beijing Road, Qingshanhu District, Nanchang 330029, China; Department of Radiation Oncology, Jiangxi Cancer Hospital, 519 East Beijing Road, Qingshanhu District, Nanchang 330029, China; Department of Electronic Information Engineering, 999 Xuefu Dadao, Honggutan District, Nanchang 330031, China; Department of Radiation Oncology, Jiangxi Cancer Hospital, 519 East Beijing Road, Qingshanhu District, Nanchang 330029, China

**Keywords:** craniospinal irradiation, feasibility dose–volume histogram, multi-isocenter, auto-planning, VMAT

## Abstract

This study aims to propose a novel treatment planning methodology for multi-isocenter volumetric modulated arc therapy (VMAT) craniospinal irradiation (CSI) using the special feasibility dose–volume histogram (FDVH)-guided auto-planning (AP) technique. Three different multi-isocenter VMAT -CSI plans were created, including manually based plans (MUPs), conventional AP plans (CAPs) and FDVH-guided AP plans (FAPs). The CAPs and FAPs were specially designed by combining multi-isocenter VMAT and AP techniques in the Pinnacle treatment planning system. Specially, the personalized optimization parameters for FAPs were generated using the FDVH function implemented in PlanIQ software, which provides the ideal organs at risk (OARs) sparing for the specific anatomical geometry based on the valuable assumption of the dose fall-off. Compared to MUPs, CAPs and FAPs significantly reduced the dose for most of the OARs. FAPs achieved the best homogeneity index (0.092 ± 0.013) and conformity index (0.980 ± 0.011), while CAPs were slightly inferior to the FAPs but superior to the MUPs. As opposed to MUPs, FAPs delivered a lower dose to OARs, whereas the difference between FAPs and CAPs was not statistically significant except for the optic chiasm and inner ear_L. The two AP approaches had similar MUs, which were significantly lower than the MUPs. The planning time of FAPs (145.00 ± 10.25 min) was slightly lower than that of CAPs (149.83 ± 14.37 min) and was substantially lower than that of MUPs (157.92 ± 16.11 min) with *P* < 0.0167. Overall, introducing the multi-isocenter AP technique into VMAT-CSI yielded positive outcomes and may play an important role in clinical CSI planning in the future.

## INTRODUCTION

Craniospinal irradiation (CSI) targets the entire central nervous system axis, which plays a pivotal role in the treatment of malignant neoplasms with a high risk of cerebrospinal fluid dissemination, such as primitive neuroectodermal tumors, medulloblastoma, pineoblastoma and ependymoma [1–3]. Due to the large target volume and complex-shaped anatomy, treatment planning for CSI remains challenging to cover the large planning target volume (PTV) with the limited field size of common linear accelerator (LINAC) while maintaining the PTV uniformity and avoiding over- and/or underdosage in the junction areas [4, 5]. The traditional 3D forward-planned CSI technique consists of two lateral opposed fields for the cranial and adjusts one or more abutting posterior fields for the spine [6, 7]. The dose feathering method of shifting the treatment field junctions with an extension of 0.5–1 cm weekly has been adopted to neutralize hot spots and cold spots in the junctions [8]. Although the conventional technique has been in widespread use, it still encounters significant challenges in the field of robust dosimetry, reproducible positioning and serious radiation-associated side effects [9]. To overcome these limitations, advanced treatment modalities, such as intensity-modulated radiotherapy (IMRT), volumetric modulated arc therapy (VMAT), TomoTherapy and particle beam therapy, have been explored to improve plan conformity and clinical benefits [10–16]. In parallel, the practice of field-junction strategies evolved from simple junction-moving to sophisticated approaches by tapping the potential of dose sculpting within inverse planning optimization (IPO), such as ‘hybrid-junction’ [7], ‘jagged-junction’ [17], ‘gradient-optimization’ [18, 19], ‘overlap-technique’ [20–22] and ‘staggered overlap’ [23].

In recent years, by optimizing any number of overlapped arcs concurrently and by defining the properties of each arc separately, junction-VMAT with LINAC has become an established method for CSI and has the advantages of a more homogeneous dosage and better sparing of organs at risk (OARs) [24–26]. However, meticulous planning for smooth field junctions complicates the IPO-based approach. Although this method is effective, physicists may not be able to obtain a perfect solution for the multi-isocenter VMAT-CSI (miVMAT-CSI) in a limited time. The process of repeated trial and error during plan setup and reoptimization would spend much effort on manual parameter tuning [18, 23, 27]. During the manual optimization process, artificial tuning structures, including dummy structures, additional rings and hot/cold spots, are frequently introduced to achieve a proper balance between target coverage and OARs sparing. With limited clinical resources, one of the major challenges of miVMAT-CSI planning is the enormous variation in the quality of plans due to the complexity of CSI and varied skills among planners.

Tremendous technology advancements for RT have enabled the creation and delivery of more intelligent RT plans over the past few years. Deliberately mimicking the behavior of experienced planners, auto-planning (AP) algorithms, including Auto-Plan (Philips Medical Systems, Best, The Netherlands) [28], Rapid-Plan (Varian Medical Systems, Palo Alto, CA) [29] and Multi-Criteria Optimization (RaySearch Laboratories, Stockholm, Sweden) [30], have been developed to accelerate the treatment planning process and drastically improve the planning efficiency. During the optimization process, the AP module reduces manual intervention and interoperator variability. However, AP quality is highly dependent on the initial requirements and planner experience. In other words, AP does not remove the uncertainty about whether clinical goals will be achieved [30–32]. Purposely designed to eliminate the reliance on manually initialized inputs and to provide therapy plans tailored to the unique clinical requirements of a specific patient, PlanIQ Feasibility software (Sun Nuclear, Melbourne, FL, USA) was seamlessly integrated into the recently released Pinnacle Evolution system [31–33]. Based on the valuable assumption for the dose fall-off from the prescribed dose at the target boundary, PlanIQ provides the best possible OARs sparing for a particular patient. Allowing the planning parameters recommended by PlanIQ to be personalized according to the unique anatomical geometry significantly improves the treatment plan quality without many optimizations. The feasibility dose–volume histogram (FDVH) quantitative evaluation tool implemented in PlanIQ identifies four DVH regions, i.e. impossible region, difficult region, challenging region and probable region, which enables visualization of superior outcomes prior to actual optimization, thus providing meaningful information for decision-making for the medical physicist.

In the present study, we specifically sought to develop a novel approach for miVMAT-CSI planning by combining multi-isocenter VMAT and the special FDVH-guided AP (FAP) technique and to demonstrate its feasibility and advantages for improving planning quality and efficiency concerning manually optimized plans (MUPs). Three different miVMAT-CSI plans were created and compared, including MUPs, conventional AP plans (CAPs) and special FAP plans. In summary, our major contributions include the following. First, based on the problems encountered in clinical practice mentioned above, we proposed a new framework for miVMAT-CSI planning by using innovative FDVH-guided AP technique and verified its feasibility and effectiveness. Statistical results showed that the two AP (CAP and FAP) methods, especially for FAPs, can significantly spare the OARs while maintaining target conformity compared with the MUPs. To the best of our knowledge, this is the first attempt for a combination of multi-isocenter VMAT and FAP techniques in the field of CSI planning, which is promising for decreasing the variability and complexity compared to MUPs planning. Second, the present approach provides a new strategy for complex multi-isocenter CSI planning and significantly lowers the technological threshold that benefits junior physicists. Third, detailed experiments were performed through qualitative and quantitative evaluations in terms of visualizations, dosimetric metrics, planned monitor units (MUs) and planning time for MUPs, CAPs and FAPs.

## MATERIALS AND METHODS

### Patients and simulation

In sum, 36 treatment plans for 12 patients treated with CSI were chosen for retrospective planning using the multi-isocenter VMAT technique. The patient group consisted of seven male and five female patients with a median patient age of 10 years (range, 6–19 years). The mean PTV length in the craniocaudal (CC) direction was 65.1 cm (ranging from 51.7 to 87.3 cm), which requires two or three isocenters for irradiation via Elekta Infinity LINAC. The detailed clinical characteristics of these patients are summarized in Table 1. The head and neck of the patients were immobilized with individual thermoplastic masks in a head-first supine position, with arms resting suitably alongside the body, and the torso was immobilized with a vacuum fixation cushion. Simulation CT images were acquired with 3-mm slice thicknesses. A CT scan was derived from the cranial vertex to the proximal femur, and images were transferred into the Pinnacle treatment planning system (TPS) for structure contouring. The clinical target volume (CTV) covering the whole brain, meninges and spinal canal was determined by a senior radiation oncologist according to the clinical guideline [34, 35]. The final PTV was generated by uniform expanding cranial CTV and spinal CTV with 3- and 5-mm margins, respectively. The lens, optic nerve, optic chiasm, inner ears, thyroid, pituitary, parotid glands, esophagus, trachea, heart, lungs, stomach, kidneys, small bowels and liver were contoured as OARs for comparison.

**Table 1:** Characteristics of 12 patients enrolled in this study

Patient	Sex	Age	PTV length (cm)	Isocenters	PTV volume (cc)	Pathology
1	Female	9	71.4	3	2373.10	Medulloblastoma
2	Male	12	65.4	2	2597.08	Medulloblastoma
3	Male	10	69.0	3	1974.14	Medulloblastoma
4	Male	17	81.6	3	2349.32	Pineoblastoma
5	Male	7	57.0	2	2064.23	Medulloblastoma
6	Female	8	60.0	2	1864.53	Medulloblastoma
7	Male	6	53.7	2	2060.76	Medulloblastoma
8	Female	10	64.5	2	1942.18	Medulloblastoma
9	Female	7	57.5	2	1967.87	Medulloblastoma
10	Male	19	79.2	3	2377.29	Seminoma
11	Male	11	64.2	2	2603.19	Medulloblastoma
12	Female	15	65.4	2	2184.1	Medulloblastoma

### Treatment planning

**Fig. 1:**
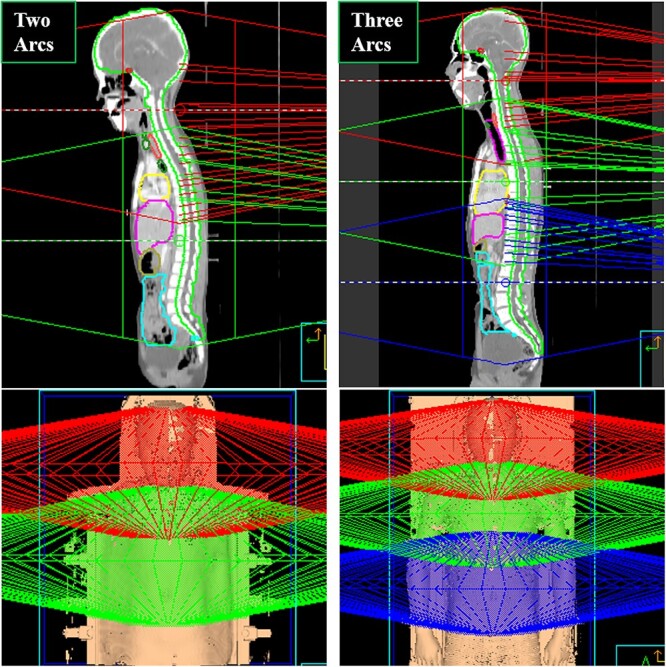
Beam’s eye view of beam arrangements and delineated structures for miVMAT-CSI.

Due to the overlength of the PTV, the two/three isocenters separated longitudinally from 20 to 28 cm, and each overlapping region was properly defined between 10 and 15 cm for adjacent arcs to produce a homogeneous dose transition. To simplify the positioning and minimize the potential risk associated with couch movement error, the coordinates of the isocenters differed only in the CC direction, and the positions were suitably determined according to different PTV lengths. The delineated structures and beam arrangement are shown in Fig. 1. For the cranial PTV, one 360° arc was used to cover the whole brain and partial spinal PTV. Another one or two full arcs were used to cover the remaining spinal PTV. The collimator and couch rotation were set to 0°. All treatment plans were optimized using a dose calculation grid size of 2 × 2 × 2 mm and have prescribed a dose of 36 Gy (20 fractions); junction-VMAT with overlap-technique has been used in this work.

**Fig. 2:**
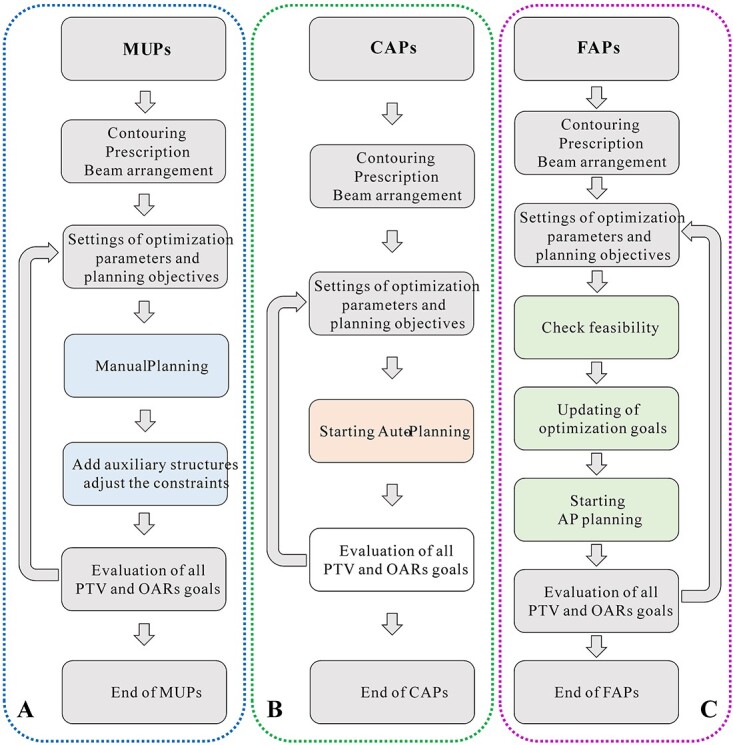
The clinical workflow for treatment planning. A:MUPs; B:CAPs; C:FAPs.

The clinical workflow for MUPs, CAPs and FAPs is shown schematically in Fig. 2. Built on clinical requirements, experience and customization, the initial optimization parameters and planning objectives of MUPs were predefined by one senior medical physicist. Considering that the super long target required efforts to ensure appropriate dose coverage and homogeneity, all plans were optimized, giving high weight for the PTV and smaller weight for the OARs. Typically, to achieve acceptable CSI plans, the planner needs to introduce auxiliary structures frequently and repeatedly to adjust the constraints. Additionally, manual normal tissue objectives and rings were utilized to limit hot spots or control dose fall-off outside the PTV. In this work, guided by identified patient-specific structural possibilities. The physicist needs to manually adjust the predefined objectives four to seven times to meet the final clinical requirements.

For CAPs, we created the template with the same beam configuration (isocenter positions, overlapping lengths, collimator angle and couch angle) and initial objectives of previous MUPs. It is worth noting that the current AP script installed in Pinnacle3 Version 9.10 cannot be used for designing multi-isocenter VMAT, and the multiple arcs from different isocenters should be manually deployed in the field setting interface after importing the traditional AP template. According to the configuration file from the inverse optimizer plugin installed in Pinnacle, the AP module enables planners to automatically adjust objectives and to create tuning regions of interest (ROIs) iteratively to best meet user-defined clinical goals during the optimization process. Furthermore, the optimizer plugin checks the hard constraint list and still optimizes until it is satisfied.

Figure 2C shows the clinical workflow for FAPs. Based on the valuable assumption for the dose fall-off from the prescribed dose at the target boundary, the FDVH from PlanIQ provides the possibility of OARs sparing according to the specific anatomical geometry. FAPs had identical beam arrangements and optimization settings with CAPs. Specifically, the initial settings for planning goals were first imported by referring to CAPs, then checking the feasibility for all OARs and finally updating the goals according to FDVH. The detailed settings were as follows: the beam energy was 6 MV, the tuning balance was 11%, the dose fall-off margin was 2.6 cm, the control point spacing was 4°, the max iteration was 90, the convolution iteration was 12, the minimum segment area was 4 cm^2^ and the minimum segment MUs was constrained to 2. After AP optimization, an additional one to three manual tweaks were performed. Table 2 lists the settings of optimization goals, compromise and priority by the planner. Most of the optimization functions of FAPs were significantly lower than those of CAPs, which enables the AP algorithm to be more restricted for optimization and to thus minimize the dose to OARs.

**Table 2:** OARs optimization parameters in MUPs, CAPs and FAPs

ROIs	Type	Dose (cGy)		Volume	Priority	Compromise	Feasibility
	MUPs/CAPs/FAPs	MUPs/CAPs	FAPs	CAPs/FAPs	CAPs/FAPs	CAPs/FAPs	FAPs
Lens	Max dose	600	500–600		High	No	Challenging
Optic nerves	Max dose	3400	3400–3500		Medium	No	Difficult
Optic chiasm	Max dose	3500	3500–3600		Low	Yes	Impossible
Inner ears	Max dose	3500	3500–3600		Low	Yes	Impossible
Parotid glands	Mean dose	900	700–800		Medium	Yes	Probable
Parotid glands	Max DVH	1000	600–900	50%	Medium	Yes	Probable
Stomach	Max dose	1200	1000–1200		Low	No	Probable
Pituitary	Max dose	3500	3500–3600		Low	Yes	Impossible
Thyroid	Max DVH	800	600–700	20%	Low	Yes	Probable
Esophagus	Max DVH	1200	900–1100	20%	Low	Yes	Probable
Trachea	Max dose	1000	700–900	20%	Low	Yes	Probable
Heart	Mean dose	800	600–800		Low	Yes	Probable
Lung	Max DVH	600	500–600	65%	Medium	Yes	Probable
Lung	Max DVH	900	800–900	30%	Medium	Yes	Probable
Lung	Max DVH	1200	1000–1200	20%	Medium	Yes	Probable
Lung	Mean dose	1000	900–1000		Medium	Yes	Probable
Kidneys	Max DVH	800	500–800	33%	Medium	No	Probable
Kidneys	Mean dose	600	500–600		Medium	No	Probable
Liver	Mean dose	800	600–800		Low	Yes	Probable
Liver	Max DVH	600	500–600	50%	Low	Yes	Probable
Small bowels	Mean dose	800	700–800		Low	Yes	Probable

### Plan evaluation and analysis

All plans were reviewed by the senior radiation oncologist and considered acceptable if *D*_98%_ (*D_x_* means dose received by the percentage of the volume) of PTV was >95% of the prescribed dose. For OARs dose goals, as long-term survival improves, many investigations have indicated that CSI may cause severe side effects, such as hypothyroidism, scoliosis, endocrine, fertility dysfunction and even the occurrence of secondary malignancies, especially for children and adolescent patients [34–40]. Therefore, the dose for OARs should be controlled as low as possible to minimize the side effects.

Dosimetric evaluation metrics, including *D*mean and *D*max (the mean dose and maximum dose to target volume), *D*_2_ and *D*_98_, *V*_95_ and *V*_100_ (*V_x_* represents the volume receiving at least *x*% of the prescription dose) of the PTV, were calculated and compared. The homogeneity index (HI), conformity index (CI) and DVH analysis for each plan were evaluated. HI was expressed in terms of (*D*_2%_ − *D*_98%_)/*D_p_* × 100%, where *D_p_* is the prescribed dose [41]. A value of HI = 0 indicates an ideal plan with excellent homogeneity. CI was defined as the ratio between the PTV volume covered by the prescribed dose and the volume of the PTV, and a CI specifying an index value closer to 1 indicates better conformity [42]. Dosimetric parameters of the OARs include the *D*max to the lens, optic nerves, thyroid, pituitary, inner ears, optic chiasm and stomach and the *D*mean to the parotid glands, esophagus, trachea, heart, lungs, small bowels, kidneys and liver. The volumes of the lungs receiving 5 Gy (*V*_5Gy_) and 10 Gy (*V*_10Gy_) were also evaluated. Additionally, the planned MUs and planning time were calculated to assess the efficiency. The planning time was approximately recorded and was defined as the time from the beginning of planning to the end of optimization/dose calculation per fraction. All comparisons were performed using SPSS software (version 19.0, IBM). A paired Wilcoxon sign-rank test was carried out to determine statistical significance between plans. A Bonferroni correction was used in order to account for multiple hypothesis testing (*P* < 0.0167 was needed for significance).

## RESULTS

**Fig. 3:**
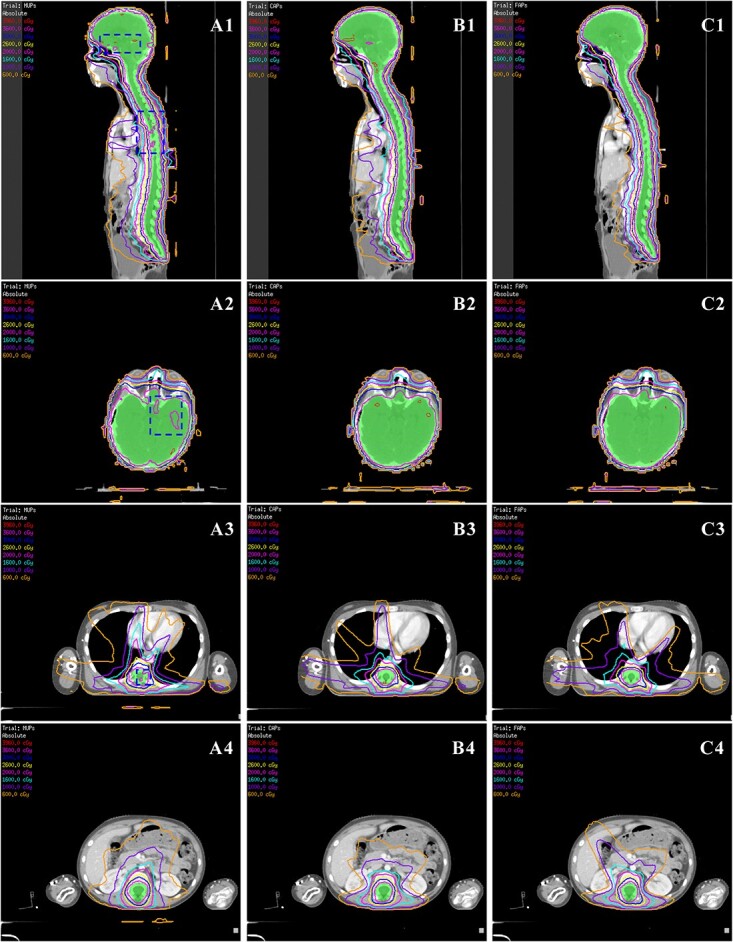
Sagittal and transversal dose distributions. A1–A4 denote MUPs, B1–B4 denote CAPs and C1–C4 denote FAPs.

**Fig. 4:**
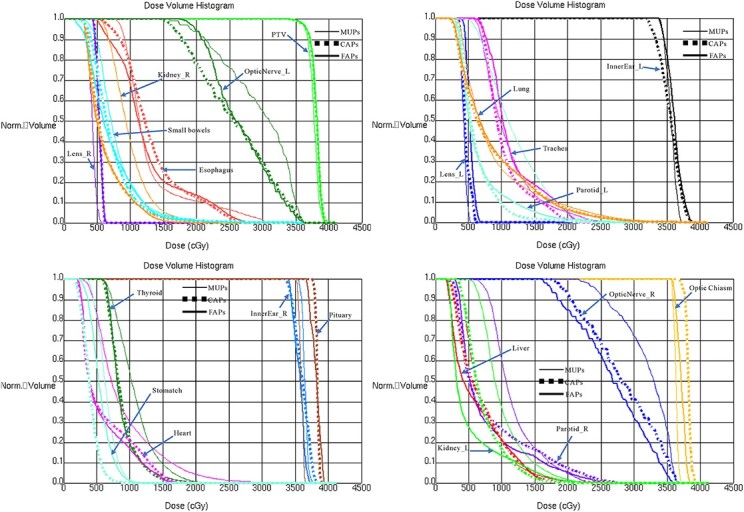
Comparison of DVHs for the PTV and OARs in MUPs, CAPs and FAPs.

The transverse and sagittal dose distributions for different regions of one representative patient, with corresponding cumulative DVHs for the PTV and OARs, are shown in Figs 3 and 4. The hot spots and cold spots in the MUPs were slightly higher and lower than those in the AP plans, respectively. The isodose lines were more conformal and constrictive to the target for FAPs compared to CAPs and MUPs in terms of visual inspection. Especially for the sagittal views, a steeper dose gradient was observed, which further demonstrated improved conformity in PTV and supported the reduction of OARs values. For better illustration, the dosimetric differences of the target volume and individual organs were presented in a DVH for this patient (Fig. 4).

To quantitatively evaluate the dosimetric performance of the three treatment modalities, Table 3 shows an overview of the numerical findings from the average DVH analysis on the PTV and OARs. The target dose conformity of MUPs (0.969 ± 0.014) was inferior to the other two methods; the FAPs (0.980 ± 0.011) achieved the best CI, whereas the CAPs (0.977 ± 0.012) were slightly better than the MUPs. Similar findings could be observed for other DVH metrics of HI, *D*_98_, *V*_95_ and *V*_100_. In terms of CAPs and FAPs, a slight improvement in PTV was observed for FAPs. As summarized in Table 3, the CAPs and FAPs significantly reduced the dose for most OARs, and FAPs yielded a smaller dose to all organs compared with CAPs; no significant difference was observed among them except for optic chiasm and inner ear_L. In addition, the maximum doses to the lens_R and bilateral optic nerves for MUPs were comparable with those for CAPs and FAPs. Overall, these findings indicated that AP methods were able to lower doses to OARs while maintaining PTV coverage. As displayed in Table 4, a significantly reduced number of MUs in the AP plans was observed. The average MUs for MUPs, CAPs, and FAPs were 900.7, 781.7 and 765.7, respectively. CAPs and FAPs had almost similar MUs. The total planning time of MUPs increased by 8.09 min and 12.92 on average compared to CAPs and FAPs.

**Table 3:** Quantitative evaluation on the average and standard deviation values of DVH metrics for PTV and OARs among MUPs, CAPs and FAPs

Structures	DVH metrics	MUPs	CAPs	FAPs	*p*1	*p*2	*p*3
PTV	*D*mean (Gy)	37.69 ± 0.19	38.03 ± 0.09	38.16 ± 0.20	0.004	0.010	0.041
	*D*_2_ (Gy)	39.23 ± 0.35	39.52 ± 0.13	39.44 ± 0.18	0.013	0.028	0.119
	*D*_98_ (Gy)	35.74 ± 0.40	35.98 ± 0.45	36.13 ± 0.42	0.050	0.003	0.017
	*V*_95_ (%)	99.63 ± 0.29	99.66 ± 0.31	99.67 ± 0.29	0.059	0.050	0.683
	*V*_100_ (%)	96.93 ± 1.43	97.67 ± 1.15	98.02 ± 1.01	0.099	0.005	0.002
	CI	0.969 ± 0.014	0.977 ± 0.012	0.980 ± 0.011	0.099	0.004	0.008
	HI	0.097 ± 0.014	0.098 ± 0.012	0.092 ± 0.013	0.937	0.102	0.017
Parotid_L	*D*mean (Gy)	12.23 ± 0.86	8.35 ± 2.58	8.47 ± 2.06	0.002	0.002	0.937
Parotid_R	*D*mean (Gy)	12.53 ± 1.00	8.34 ± 1.60	8.11 ± 2.09	0.003	0.002	0.272
Optic Chiasm	*D*max (Gy)	39.19 ± 0.31	38.94 ± 0.51	37.65 ± 0.44	0.480	0.002	0.002
Optic Nerve_L	*D*max (Gy)	35.71 ± 1.55	35.69 ± 0.79	35.09 ± 1.97	0.754	0.084	0.158
Optic Nerve_R	*D*max (Gy)	36.19 ± 0.50	36.14 ± 1.80	35.98 ± 1.69	0.695	0.999	0.814
Inner Ear_R	*D*max (Gy)	38.23 ± 0.79	37.80 ± 0.60	37.56 ± 0.49	0.136	0.071	0.239
Inner Ear_L	*D*max (Gy)	38.63 ± 0.49	38.63 ± 0.75	37.88 ± 0.31	0.929	0.005	0.012
Thyroid	*D*mean (Gy)	10.72 ± 0.54	6.51 ± 0.78	6.44 ± 0.93	0.002	0.002	0.530
Pituitary	*D*max (Gy)	38.45 ± 0.53	38.36 ± 0.73	38.22 ± 0.49	0.421	0.542	0.751
Lens_L	*D*max (Gy)	5.67 ± 0.44	5.43 ± 0.41	5.28 ± 0.44	0.158	0.033	0.169
Lens_R	*D*max (Gy)	5.63 ± 0.47	5.02 ± 0.47	5.19 ± 0.43	0.028	0.060	0.209
Stomach	*D*max (Gy)	12.08 ± 1.09	10.43 ± 2.98	10.00 ± 2.49	0.028	0.008	0.328
Esophagus	*D*mean (Gy)	11.87 ± 0.41	9.70 ± 2.11	8.91 ± 1.92	0.010	0.002	0.209
Lungs	*D*mean (Gy)	8.39 ± 0.60	8.15 ± 1.35	7.81 ± 1.27	0.638	0.060	0.209
	*V*_5Gy_ (%)	71.21 ± 5.71	65.52 ± 14.77	63.00 ± 13.80	0.028	0.117	0.433
	*V*_10Gy_ (%)	25.84 ± 6.90	24.96 ± 6.26	24.27 ± 2.44	0.367	0.347	0.638
Kidney_L	*D*mean (Gy)	9.31 ± 0.76	5.40 ± 1.48	5.27 ± 1.39	0.002	0.002	0.784
Kidney_R	*D*mean (Gy)	9.15 ± 0.76	4.73 ± 1.08	4.69 ± 1.60	0.002	0.002	0.999
Liver	*D*mean (Gy)	6.98 ± 0.38	6.36 ± 0.74	6.26 ± 0.84	0.006	0.003	0.695
Small bowels	*D*mean (Gy)	8.05 ± 0.74	7.27 ± 1.36	7.21 ± 1.37	0.015	0.015	0.410
Trachea	*D*max (Gy)	10.96 ± 0.66	8.33 ± 1.91	8.30 ± 1.81	0.003	0.004	0.969
Heart	*D*mean (Gy)	8.46 ± 1.24	6.13 ± 1.14	5.84 ± 1.22	0.003	0.002	0.289

Notes: DVH = dose–volume histogram, MUPs = manually plans, *p*1 = MUPs vs CAPs, *p*2 = MUPs vs FAPs, *p*3 = CAPs vs FAPs.

## DISCUSSION

Currently, manual-based treatment planning is still the mainstream in clinical practice, whereas the approach of manually setting parameters, tweaking clinical goals and assigning importance weights remains very time-consuming and labor-intensive [29–31]. During the optimization process, artificial tuning structures, including dummy structures, additional rings and ROIs of hot/cold spots are frequently introduced to achieve a proper balance between target coverage and OARs sparing. With limited clinical resources, one of the major challenges of VMAT-CSI planning is the large variation in the quality of plans due to the complexity of CSI and varied skills among planners. Under limited time pressure, even the most experienced planner may not obtain the expected CSI plan. The speed, quality and consistency of a manually designed plan depend heavily on the experience of the planners. It is essentially impossible for physicians/dosimetrists to know whether optimization attempts have minimized the OARs dose to the lowest attainable level within an acceptable time frame, which is another major challenge. To overcome the limitations of miVMAT-CSI planning, in this work, a novel implementation of the multi-isocenter AP approach for VMAT-CSI was presented, and its operability and advantages with respect to previously manually optimized plans in terms of planning quality and efficiency were demonstrated. We found that the dosimetric performance of FAPs was slightly superior to that of CAPs and resulted in significantly better benefits than MUPs for most OARs. Additionally, we have shown that both FAPs and CAPs were superior to MUPs in terms of conformity, *D*_98_, *V*_95_, *V*_100_ and CI of PTV. The statistics in Tables 2–4 show a notable improvement in the quality of the two AP methods compared with MUPs. This improvement is also reflected in the results shown in Figs 3 and 4.

**Table 4:** MUs and planning time

Parameter	MUPs	CAPs	FAPs	*p*1	*p*2	*p*3
MUs	900.7 ± 156.5	781.7 ± 142.6	765.7 ± 123.9	0.002	0.002	0.583
Planning time (min)	157.92 ± 16.11	149.83 ± 14.37	145.00 ± 10.25	0.003	0.005	0.061

Notes: MUs = monitor units; *p*1 = MUPs vs CAPs, *p*2 = MUPs vs FAPs, *p*3 = CAPs vs FAPs.

Previously, various AP approaches have been investigated to automate and speed up the planning process while maintaining or improving the consistency and quality of RT plans. It utilizes a progressive optimization algorithm to systematically add planning objectives and artificial contours and automatically adjust goal sheets and weights based on the expected target coverage and OARs sparing. The proliferation and commercial availability of these solutions have enhanced the decision-making power of clinicians and effectively reduced the variation in operator intervention. However, the AP technique has encountered some important limitations, as illustrated by Ouyang *et al*. [43] and Esposito *et al*. [44]. In general, the selection of initial optimization parameters directly affects the quality of the final plan. However, these generic initial parameters are not suitable for all situations and need to be manually modified through trial and error. In the actual practice of RT planning, it is challenging to provide optimal target coverage while maximizing OARs sparing. If physicians/dosimetrists have insight into how much OARs dose can be reduced without compromising target coverage with preoptimization, this would help the planners to define the clinical goals, which eventually result in a better dosimetric outcome.

As a commercially available software, PlanIQ provides *a priori* estimation on the achievability of dose reduction in DVH for OARs, and the fine-tuning process performed by PlanIQ is far quicker than manual trial-and-error planning. Just recently, PlanIQ has been integrated into the TPS system. By taking advantage of the FDVH function in PlanIQ, the planner knew from the beginning of planning whether these prescription requirements could be achieved, which greatly saved planning time. As described previously, feedback information from FDVH can assist planners in visualizing how the ideal prescription, patient-specific anatomy, critical structures and target volumes interact within dose-junction areas. Fried *et al*. reported that using FDVH in VMAT planning for patients with head and neck cancer significantly reduced OARs doses by an average of 200–700 cGy while maintaining PTV coverage [31]. Perumal *et al*. showed that AP significantly reduced the OAR doses in various anatomic sites under the guidance of PlanIQ software [32]. Xia *et al*. conducted a retrospective study on 10 lung cancer patients and found that the AP module of the Pinnacle system delivered significantly higher plan quality using the personalized plan parameters recommended by the PlanIQ feasibility tool [45]. Moreover, Shimizu *et al*. determined the acceptable *f*-value (0.22–0.26) on the FDVH of PlanIQ software for effective dose reduction of the normal lung in patients with Stage III non-small cell lung cancer [46]. Similar to other studies mentioned above, the present findings showed that the initial inputs of CAPs were dependent on MUPs, which leads to a relatively inferior approach to the protection of OARs compared to FAPs. Furthermore, benefiting from the prior goal setting of FDVH, the FAPs had a significantly lower planning time than CAPs/MUPs and yielded smaller doses to all organs. Although PlanIQ requires additional time to calculate the feasibilities, which will result in a slight increase in the single optimization time of FAPs, the optimization number of times is greatly reduced due to the excellent performance of FDVH. The results in Table 3 show that the dose deposition in most OARs was dramatically improved by using personalized parameters derived from FDVH compared to MUPs. Through detailed experimental studies, we found that the feasibility of the optic chiasma, pituitary and inner ears were always ‘Impossible’, which may be attributed to the large overlaps of the PTV with these OARs or the strict restriction of these planning objectives.

There are some potential limitations to this work. First, we did not compare our method to manual-based 3D conformal radiotherapy or the IMRT technique. Second, the number of patients was relatively small, and the conclusions can be further supported by increasing the sample size in future studies. Third, treatment plan/dose verification is not presented in the current retrospective study, and this should be done before clinical radiotherapy. Last but not least, this work focuses on exploring a multi-isocenter AP planning approach and evaluating its usefulness for improving the quality of CSI plans. We did not provide additional experiments about the effects of different overlapping lengths and setup errors. However, we consider these limitations to be of limited importance and do not significantly influence the overall findings of the study.

## CONCLUSION

In this work, we proposed a feasible solution to bridge the gap between the multi-isocenter VMAT and AP techniques. The novel implementation of multi-isocenter AP for VMAT-CSI was presented, and we demonstrated its feasibility and efficiency, which simplifies the plan optimization process while maintaining or improving quality. AP-based VMAT-CSI was almost equivalent and overall superior to manually optimized plans and may play an important role in clinical planning in the future.

## FUNDING

This work was supported in part by the ‘Science and Technology Planning Project of Jiangxi Provincial Health Commission’ (Nos. 202210054 and 202310023) and the ‘Jiangxi Provincial Natural Science Foundation’ (No. 20224BAB206070).

## CONFLICT OF INTEREST

The authors declare that they have no competing interests.

## DATA AVAILABILITY

The data underlying this article will be shared on reasonable request to the corresponding author.
